# Prognostic values of ALDOB expression and ^18^F-FDG PET/CT in hepatocellular carcinoma

**DOI:** 10.3389/fonc.2022.1044902

**Published:** 2022-12-08

**Authors:** Wenzhi Jia, Qianyun Wu, Xiaofeng Yu, Mengqin Shen, Ruixue Zhang, Jiajin Li, Li Zhao, Gang Huang, Jianjun Liu

**Affiliations:** Renji Hospital, School of Medicine, Shanghai Jiao Tong University, Shanghai, China

**Keywords:** hepatocellular carcinoma, tumor progression, prognosis, ^18^F-FDG PET/CT, ALDOB

## Abstract

**Purpose:**

The glycolytic enzyme fructose 1,6-bisphosphate aldolase B (ALDOB) is aberrantly expressed and impacts the prognosis in hepatocellular carcinoma (HCC). Hepatic ALDOB loss leads to paradoxical upregulation of glucose metabolism, favoring hepatocellular carcinogenesis. Nevertheless, the relationship between ALDOB expression and ^18^F-fluorodeoxyglucose (^18^F-FDG) uptake, and their effects on HCC prognosis remain unclear. We evaluated whether ALDOB expression is associated with ^18^F-FDG uptake and their impacts on HCC prognosis prediction.

**Methods:**

Changes in ALDOB expression levels and the prognostic values in HCC were analyzed using data from The Cancer Genome Atlas (TCGA) database. Ultimately, 34 patients with HCC who underwent ^18^F-FDG positron emission tomography/computed tomography (PET/CT) preoperatively were enrolled in this retrospective study. ALDOB expression was determined using immunohistochemistry (IHC) staining, and the maximum standardized uptake value (SUVmax) of HCC was calculated from the ^18^F-FDG PET/CT scans. The relationship between ALDOB expression and SUVmax was examined, and their impacts on overall survival were evaluated using Cox proportional hazards models and Kaplan–Meier survival analysis. ALDOB overexpression in HUH7 and 7721 cells was used to analyze its role in tumor metabolism.

**Results:**

According to TCGA database, the ALDOB mRNA level was downregulated in HCC compared to normal tissue, and significantly shortened overall survival in HCC patients. ALDOB protein expression was similarly decreased in IHC findings in HCC than that in adjacent normal tissues (P<0.05) and was significantly associated with tumor size (P<0.001), high tumor-node-metastasis stage (P=0.022), and elevated SUVmax (P=0.009). ALDOB expression in HCC was inversely correlated with SUVmax (r=-0.454; P=0.012), and the optimal SUVmax cutoff value for predicting its expression was 4.15. Prognostically, low ALDOB expression or SUVmax ≥3.9 indicated shorter overall survival time in HCC. Moreover, COX regression analysis suggested high SUVmax as an independent prognostic risk factor for HCC (P=0.036). HCC patients with negative ALDOB expression and positive SUVmax (≥3.9) were correlated with worse prognosis. ALDOB overexpression in HCC cells significantly decreases ^18^F-FDG uptake and lactate production.

**Conclusion:**

SUVmax in HCC patients is inversely correlated with ALDOB expression, and ^18^F-FDG PET/CT may be useful for ALDOB status prediction. The combined use of ALDOB expression and ^18^F-FDG PET/CT data can provide additional information on disease prognosis in HCC patients.

## Introduction

Hepatocellular carcinoma (HCC) is derived from hepatocytes and is the most common primary malignant tumor of the liver and the seventh most common cancer worldwide. In 2020, more than 900,000 new cases of liver cancer were reported all over the world, resulting in approximately 800,000 deaths, with incidence and mortality rates continuing to increase (1). Over 80% of patients with HCC have already developed intrahepatic or distant metastases upon initial diagnosis (2). Despite the use of various treatments, such as surgery, ablation, transcatheter arterial chemoembolization, and targeted therapy, the frequent postoperative recurrence or metastasis of HCC results in high mortality rates, with an incidence/mortality ratio close to 1.0 and a 5-year survival rate of 50% (3). Clinicopathological features (e.g., tumor size, cirrhosis, hepatitis B virus [HBV] infection, and vascular invasion) are routinely used to predict the prognosis of HCC. To reduce the HCC-associated mortality, early diagnosis and risk classification contribute to the selection of appropriate treatments to improve prognosis. Therefore, reliable biomarkers for predicting the prognosis of HCC are urgently needed.

Aldolase B (ALDOB), also known as fructose-bisphosphate aldolase or liver-type aldolase, is one of three fructose 1,6-bisphosphate aldolase isozymes that catalyze the cleavage of fructose 1,6-biphosphate into glyceraldehyde 3-phosphate and dihydroxyacetone phosphate (DHAP) and fructose 1-phosphate into glyceraldehyde and DHAP (4). ALDOB is an important enzyme in glucose and fructose metabolism and plays a key role in glycolysis and gluconeogenesis (5). The liver, a key metabolic organ, participates in various metabolic activities in the body. The role of ALDOB in the liver has received increasing attention. Multiple studies have shown that both mRNA and ALDOB protein levels are significantly downregulated in patients with HCC than in those with paracancerous liver tissue, and its downregulation is associated with aggressive characteristics and poor HCC prognosis (6, 7). Further studies showed that ALDOB could directly bind to and inhibit glucose-6-phosphate dehydrogenase, thereby downregulating the pentose phosphate pathway to suppress hepatocellular carcinogenesis (8). Tao et al. found that ALDOB inhibits HCC cell line invasion partly through ten-eleven translocation 1 expression (6). Consequently, downregulation of ALDOB in HCC contributes to metabolic dysregulation, leading to carcinogenesis, invasion, and metastasis. Therefore, ALDOB may be a potential prognostic biomarker and therapeutic target in HCC. The ability to predict its expression using noninvasive imaging modalities would be clinically significant, yet this has not been achieved.

^18^F-fluorodeoxyglucose positron emission tomography/computed tomography (^18^F-FDG PET/CT) is the most widely used noninvasive functional imaging modality for the diagnosis, staging, metastasis and recurrence detection, and treatment response evaluation in various tumors, as well as the assessment of tumor biological behavior (9–11). Accelerated FDG uptake, based on enhanced glucose metabolism in cancer cells, is a hallmark of tumors and can be quantified using the maximum standardized uptake value (SUVmax). Although ^18^F-FDG PET/CT is limited in the diagnosis of HCC due to differentiation and metabolic heterogeneity, in terms of prognosis power, ^18^F-FDG PET/CT is known to be effective (12–15). Additionally, previous studies have shown an association between FDG uptake and aggressive biomarkers in various tumors, which is beneficial for selecting clinical medications and improving prognosis. However, whether ^18^F-FDG PET/CT predicts the status of ALDOB and whether a prognostic prediction model combining SUVmax and ALDOB expression is related to overall survival (OS) in HCC patients have not been elucidated.

Therefore, this retrospective study aimed to explore the correlation between ALDOB expression and metabolic parameters of ^18^F-FDG PET/CT in HCC. Additionally, we aimed to determine whether ALDOB expression combined with metabolic parameters could better predict the prognosis of HCC patients.

## Material and methods

### Study population

This retrospective study included 34 patients with HCC. All patients underwent ^18^F-FDG PET/CT imaging before surgery (tumor resection or liver transplantation) at Shanghai Jiao Tong University affiliated with Renji Hospital between January 2007 and February 2015. The inclusion criteria were as follows: HCC diagnosis confirmed by pathological examination of surgical specimens; comprehensive case records, including age, sex, HBV infection, α-fetoprotein (AFP) status, tumor size, tumor stage, and histologic differentiation; and sufficient tissue specimens for immunohistochemical staining. Among these, histologic differentiation degrees of HCC are subgrouped as high, medium and low, recommended by the World Health Organization (WHO) book on the “Classification of Tumours of the Digestive System”. The definitions are described below. In the well-differentiated tumor, the cells resemble mature hepatocytes with minimal to mild atypia, the cytoplasms range from abundant and eosinophilic to moderate and basophilic, and the nuclei are minimal to mild nuclear atypia. The morphology of the moderately differentiated tumors strongly suggests hepatocyte differentiation, with abundant cytoplasm and moderate nuclear atypia. The poorly differentiated tumor is morphologically diverse, similar to poorly differentiated carcinoma, with moderate to scant cytoplasm and marked nuclear pleomorphism. The exclusion criteria were as follows: inadequate pathological size for immunohistochemical analysis and incomplete records. All 34 patients met the inclusion criteria, and 28 had tumor and matched peritumor tissue immunohistochemical analysis results. Informed consent was not required for this study.

### ^18^F-FDG PET/CT imaging

All patients underwent whole-body scanning using the Biograph 64 PET/CT system (Siemens Healthineers AG, Erlangen, Germany). Blood glucose levels were measured and confirmed to be< 140 mg/dL before ^18^F-FDG intravenous injection. All patients were required to fast for at least 6 hours, and the blood glucose levels were measured to be< 140 mg/dL, followed by the injection of ^18^F-FDG at a dose of 3.7 MBq/kg. PET/CT scanning was performed immediately.

Two experienced nuclear medicine physicians evaluated PET/CT images for the quantitative analyses. Regions of interest (ROIs) were drawn based on elevated ^18^F-FDG uptake. For patients without significant ^18^F-FDG uptake, ROIs were outlined based on the CT scans. SUVmax was calculated as follows: (maximum pixel activity value within the decay-corrected region of interest [MBq/mL])/ (injected dose [MBq]/kg body weight) The two physicians were aware of the clinical information, and assessed the relevant PET/CT data, including tumor location, size, morphology, and SUVmax value.

### Immunohistochemical staining

Formalin-fixed paraffin slices (thickness, 5 μm) were immunohistochemically stained with anti-ALDOB antibody (Abcam, Cambridge, UK). Two board-certified pathologists who were blinded to the patients’ clinical information independently performed immunohistochemical analysis. The percentage of immunoreactive areas covered by ALDOB was quantified and scored on a four-point scale according to the proportion (0-25%, 1; 26-50%, 2; 51-75%, 3; and 76-100%, 4). Similarly, the staining intensity was scored on a four-point scale (from 0 to 3). Tissues with >1% staining for ALDOB were considered ALDOB positive. We calculated the final ALDOB expression score by adding the two aforementioned scores.

### Gene expression correlation with survival analysis

ALDOB expression in tumor and normal tissues was determined using Gene Expression Profiling Integrative Analysis (GEPIA, http://gepia.cancer-pku.cn), which is an online open tool to explore RNA-sequencing data from HCC tumors and paracancer samples in the TCGA database. T-test was used to compare the expression levels of ALDOB in tumors and adjacent tissues. Patients’ OS was assessed using the Kaplan-Meier method, using 50% (median) as the cut-off value for the high and low expression groups. Hypothesis testing was performed using Log-rank, and P values< 0.05 were considered statistically significant.

### Cell culture

Human liver cancer cell lines Huh7 and 7721 were obtained from the Chinese Academy of Sciences and cultured in Dulbecco’s modified Eagle’s medium (DMEM) (Gibco, Grand Island, NY, USA), supplemented with 10% fetal bovine serum (Gibco), 100 μg/mL penicillin (Gibco), and 100 μg/mL streptomycin (Gibco). All cells cultured at 37°C in an incubator with 5% CO2 in a humidified atmosphere.

### Cell metabolism assay

The glucose uptake capacity in HCC cells were assessed by ^18^F-FDG values. Cells were incubated in six-well plates and transfected with either the ALDOB expression vector or a negative control vector. Treated cells were then seeded onto 12-well culture plates, and cultured in 500 µL DMEM containing ^18^F-FDG (148 kBq [4 mCi/mL]) for 1 hour at 37°C, the gamma counter assayed the radioactivity of the whole-cell lysates which were obtained by 0.1 mol/L sodium hydroxide (500 µL). Lactate levels in the medium were measured by the lactate assay kit (Lactate Assay Kit; Microdialysis, Stockholm, Sweden). Treated cells were seeded in culture dishes and incubated in DMEM without FBS for 16 h, and the culture medium was collected to measure lactate concentrations. The two above-mentioned experiments were normalized to the corresponding protein quantities using a protein assay kit (BCA Protein Assay Kit; Beyotime, Shanghai, China). All experiments were independent of each other and performed in triplicate.

### Statistical analysis

Data are presented as the mean ± standard error of the mean. The chi-squared test or Fisher’s exact test was used to assure the significance of the correlation between the binary representation of ALDOB expression and clinicopathologic characteristics. The overall survival curves about ALDOB expression and SUVmax were calculated using the Kaplan-Meier survival analysis, and comparisons were performed using the log-rank test. Spearman’s rank correlation was used to determine the association between ALDOB expression and SUVmax. The Mann–Whitney U test was used to compare ALDOB expression in terms of SUVmax and tumor size. A receiver operating characteristic curve (ROC) was used to assess the best SUVmax threshold for predicting ALDOB expression. The best SUVmax cutoff was based on the highest Youden index score. P values< 0.05 were considered statistically significant. Statistical analyses were performed using SPSS, version 21.0 (IBM Corp., Armonk, NY, USA).

## Results

### ALDOB is a prognostic biomarker for HCC

**Figure 1:**
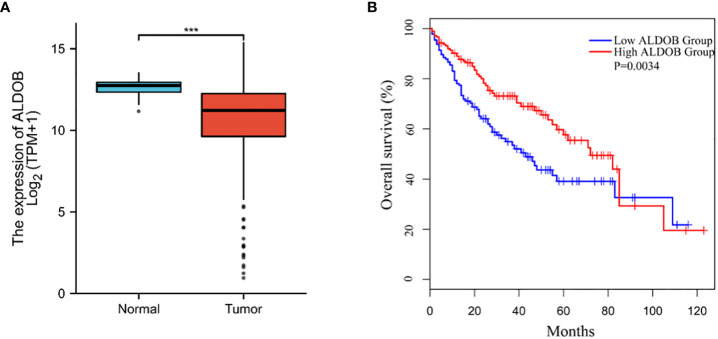
Down-regulation of ALDOB in human HCC and predicts poor prognosis. **(A)** ALDOB expression was significantly reduced in HCC compared to normal tissue. **(B)** Kaplan–Meier curve showed the correlation between ALDOB expression and overall survival of HCC patients analyzed using GEPIA. ALDOB with lower expression in the HCC samples had corresponding lower survival. ***P values< 0.001.

To assess the clinical significance of ALDOB in HCC, we queried the RNA sequencing data from The Cancer Genome Atlas (TCGA) database using GEPIA and found that ALDOB mRNA levels were downregulated in HCC (normal versus cancer)(**Figure 1A**). HCC patients with low ALDOB expression were significantly associated with poor OS (**Figure 1B**).

### ALDOB expression is correlated with clinical characteristics

**Figure 2:**
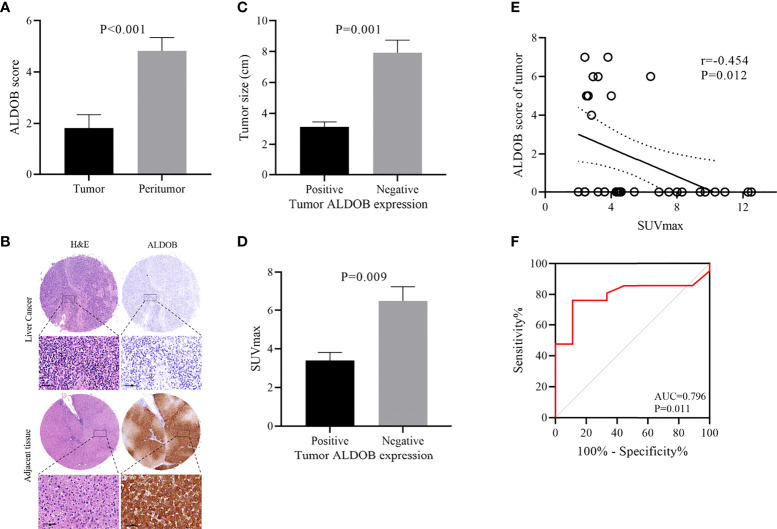
Relationship between ALDOB expression by immunohistochemistry and SUVmax in HCC patients. **(A)** Mean ALDOB score in HCC tissues was significantly lower than that in matched peritumor tissues (1.8 ± 0.5 versus 4.8 ± 0.5, P<0.001) **(B)** Histopathologic slices of peritumor and HCC tissues were stained with anti-ALDOB antibody. **(C)** ALDOB-negative tumors were obviously larger than ALDOB-positive ones (8 cm versus 3 cm; P=0.001). **(D)** SUVmax was significantly higher in ALDOB-negative tumors than in ALDOB-positive ones (6.5 ± 0.7 versus 3.4 ± 0.4; P=0.009). **(E)** Inverse correlation was found between SUVmax and tumor ALDOB expression score in HCC (r=-0.454; P=0.012). **(F)** ROC analysis of SUVmax to predict ALDOB expression. With an SUVmax of 4.15 as the threshold, sensitivity and specificity in the prediction of ALDOB expression were 76.2% and 88.9%. The area under the ROC was 0.796 (P=0.011; 95% CI 0.633~0.960).

A total of 34 HCC patients were included in this study, with an average age of 52.3 ± 1.9 years (range: 37–81 years). The average survival time was 46.0 ± 5.7 months (range: 3–108 months). The HCC tissues and corresponding peritumor tissues of 28 patients and HCC tissues of the remaining 6 patients were analyzed by immunohistochemistry (IHC). Only 26.4% (9 of 34) of HCC tissues showed positive ALDOB expression. The average ALDOB score in HCC tissues was distinctly lower than that in paired peritumor tissues (1.8 ± 0.5 and 4.8 ± 0.5, respectively; P<0.001; **Figure 2A**), which matched the former studies (6–8). A representative image of ALDOB protein expression is shown in **Figure 2B**. The relationship between ALDOB expression in HCC tissues and the clinical characteristics is shown in **Table 1**.

**Table 1:** Relationship between ALDOB expression of tumor tissues and clinical-pathologic characteristics in 34 patients with HCC.

Variable	No. of patients	ALDOB expression		P Value
		Positive	Negative	
Total patients	34	9	25	
Sex				
Male	31	9	22	0.276
Female	3	0	3	
Age (years)				
Median	–	48	51	0.539
Range	–	40~64	37~81	
α-fetoprotein status				
High	24	5	19	0.248
Low	10	4	6	
Hepatitis B virus infection				
Positive	21	5	16	0.655
Negative	13	4	9	
Hepatic sclerosis				
Presence	26	6	20	0.419
Absence	8	3	5	
Portal vein invasion				
Presence	7	0	7	0.075
Absence	27	9	18	
Tumor differentiation				
Well	2	0	2	0.143
Moderate	21	8	13	
Poor	11	1	10	
TNM Stage				
I	19	9	10	0.022
II	5	0	5	
III	6	0	6	
IV	4	0	4	
Tumor size (cm)				
Median	–	3	8	<0.001
Range	–	1.8~4.5	2~15	
SUVmax				
Median	–	2.9	5.4	0.009
Range	–	2.4~6.4	2~12.5	

Negative ALDOB expression in tumor tissue was correlated with tumor size (P<0.001), high tumor-node-metastasis (TNM) stage (P=0.022), and elevated SUVmax (P=0.009). No significant correlation was found between ALDOB expression in tumor tissues and patient age, sex, HBV infection, AFP status, hepatic sclerosis, or tumor differentiation. ALDOB-negative tumors were obviously larger than ALDOB-positive tumors (8 cm versus 3 cm; P=0.001; **Figure 2C**). Patients with a high TNM stage had negative ALDOB expression in tumor tissues, and all ALDOB-positive patients were stage I.

### Negative correlation between SUVmax and ALDOB expression in tumors

**Figure 3:**
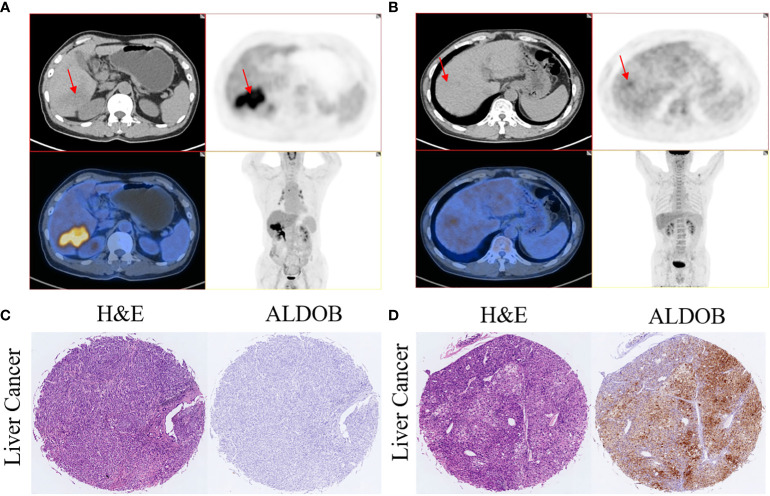
Representative images of 18F-FDG uptake and immunohistochemical staining of ALDOB in patients with HCC. **(A, C)** A 65-year-old male patient had HCC with negative ALDOB staining. 18F-FDG PET/CT scan revealed strong accumulation of 18F-FDG accumulation in the tumor lesion (SUVmax=12.5). **(B, D)** A 64-year-old male patient had HCC with positive ALDOB staining. 18F-FDG PET/CT scan did not show abnormal uptake of 18F-FDG in the tumor lesion (SUVmax=2.8).

The average SUVmax among all patients was 5.6 ± 0.6 (range: 2 to 12.5). SUVmax values were significantly higher in ALDOB-negative tumors than those in ALDOB-positive tumors (6.5 ± 0.7 versus 3.4 ± 0.4; P=0.009; **Figure 2D**). An inverse correlation was found between SUVmax and tumor ALDOB expression scores in HCC (r=-0.454; P=0.012; **Figure 2E**). To investigate the best SUVmax limens for predicting ALDOB expression in tumors, we analyzed the ROC curve and determined that the optimal SUVmax cutoff value to predict ALDOB expression was 4.15; the sensitivity and specificity to estimate ALDOB expression in tumors were 76.2% and 88.9%, respectively. The area under the curve (AUC) was 0.796 (P=0.011; 95% confidence interval [CI]: 0.633–0.960; **Figure 2F**). ^18^F-FDG PET/CT may be helpful in measuring ALDOB expression in HCC. **Figure 3** showed representative images of ALDOB IHC staining and corresponding ^18^F-FDG PET/CT scans.

### Prognostic values of SUVmax and ALDOB expression in HCC

**Figure 4:**
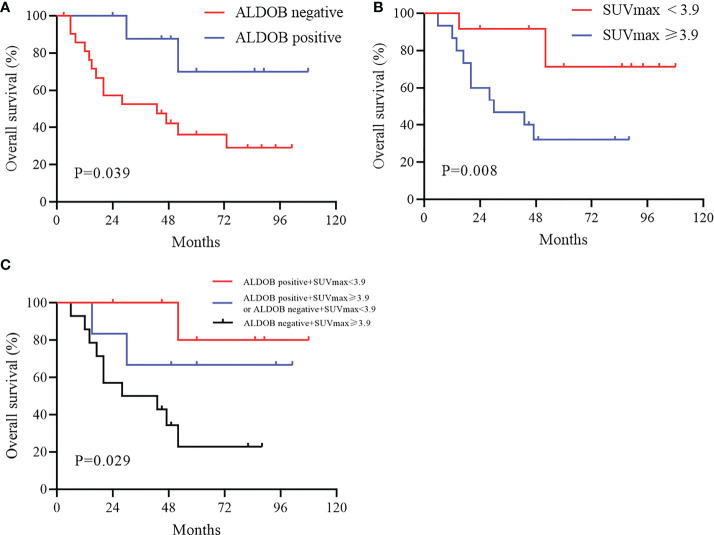
Kaplan-Meier overall survival curve in patients with HCC. **(A)** Patients with positive ALDOB expression had longer overall survival than those with negative ALDOB expression (88.5 ± 11.7 months versus 49.2 ± 8.4 months; P=0.039). **(B)** Patients with SUVmax≥3.9 had significantly shorter survival time than those with SUVmax<3.9 (43.0 ± 7.7 months versus 93.2 ± 9.5 months; P=0.008). **(C)** Elevated SUVmax (≥3.9) and negative ALDOB expression indicated worse prognosis in patients with HCC compared with two other groups (P=0.029).

Kaplan-Meier analysis revealed that patients with positive ALDOB expression had longer OS than patients with negative ALDOB expression (88.5 ± 11.7 months versus 49.2 ± 8.4 months; P=0.039; **Figure 4A**). These differences imply that ALDOB-negative HCC may manifest higher invasiveness and result in worse prognosis. ROC analysis determined an optimal SUVmax cutoff value of 3.9 for predicting survival in patients with HCC. The AUC was 0.755 (95% CI: 0.565–0.946; P=0.024), with sensitivity and specificity of 84.6% and 64.3%, respectively. Patients with SUVmax ≥3.9 had significantly shorter survival time than those with SUVmax<3.9 (43.0 ± 7.7 months versus 93.2 ± 9.5 months; P=0.008; **Figure 4B**).

All patients were divided into three groups according to SUVmax and ALDOB expression: the low SUVmax (<3.9) and ALDOB-positive; low SUVmax (<3.9) and ALDOB-negative or high SUVmax (≥3.9) and ALDOB-positive; and high SUVmax (≥3.9) and ALDOB-negative groups. Kaplan-Meier survival analysis showed that the survival time was significantly shorter in the high SUVmax (≥3.9) and ALDOB-negative group among the three groups (P=0.029; **Figure 4C**). However, no significant difference was found in survival times between the other two groups.

### Univariate and multivariate analysis of factors associated with overall survival

Univariate Cox regression analysis showed that AFP (P=0.042) and SUVmax (P=0.020) were associated with OS in patients with HCC. All factors associated with OS in the univariate analysis (P<0.1) were included in the multivariate Cox regression models. Multivariate Cox analysis showed that SUVmax (P=0.036) was the only independent factor affecting OS in HCC patients (**Table 2**).

**Table 2:** Univariate and multivariate Cox regression analysis of overall survival in patients with HCC.

Variable	Univariate analysis			Multivariate analysis		
	HR	95% CI of HR	P Value	HR	95% CI of HR	P Value
Age	1.001	0.961-1.043	0.955			
Gender	0.279	0.059-1.322	0.108			
α-fetoprotein status	3.782	1.053-13.593	0.042	2.039	0.444-9.358	0.359
Hepatitis B virus infection	0.864	0.319-2.340	0.773			
Hepatic sclerosis	5.768	0.759-43.822	0.090	3.65	0.465-28.626	0.218
Portal vein invasion	0.949	0.270-3.338	0.935			
Tumor differentiation	1.780	0.719-4.415	0.213			
TNM Stage	2.16	0.778-5.998	0.139			
Tumor size	1.09	0.982-1.211	0.107			
SUVmax	6.136	1.325-28.426	0.020	5.233	1.11-24.66	0.036
ALDOB expression	0.241	0.055-1.061	0.060	0.545	0.106-2.799	0.467

### Effect of ALDOB overexpression on cellular uptake of ^18^F-FDG

**Figure 5:**
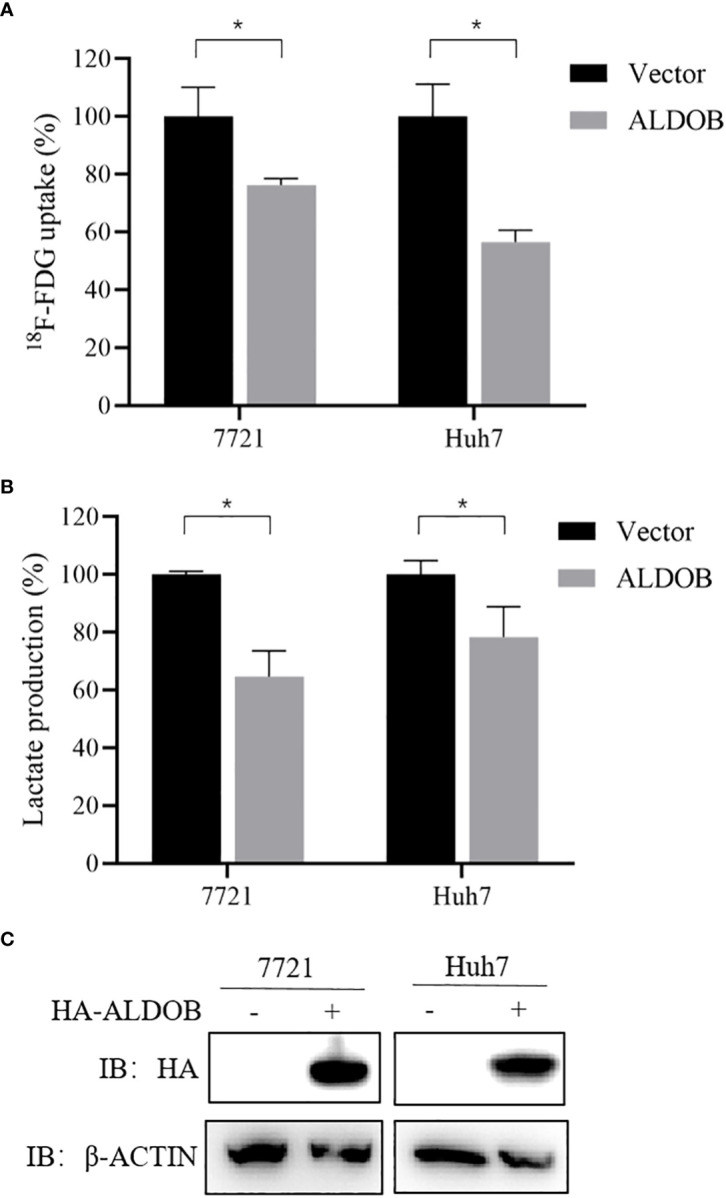
Influence of ALDOB overexpression on ^18^F-FDG uptake and lactate production in HCC cells (7721 and HuH7 cell lines). **(A)** ALDOB overexpression significantly inhibited ^18^F-FDG uptake in 7721 and HuH7 cells (P=0.017 and P=0.003). **(B)** ALDOB overexpression significantly inhibited lactate production in 7721 and HuH7 cells (P=0.013 and P=0.011). **(C)** HA-tag ALDOB and HA-VECTOR were transfected in 7721 and HuH7 cells, after 48h, the cells were lysed and protein expressions were verified by western blotting. *P values< 0.05.

Cellular ^18^F-FDG uptake and lactate production were used to evaluate the effects of ALDOB expression on glucose metabolism *in vitro*. We found that overexpression of ALDOB by 7721 and HuH7 cells significantly inhibited ^18^F-FDG uptake (P=0.017 and P=0.003, respectively; **Figure 5A**). ALDOB overexpression also reduced lactate production in these cells (P=0.013 and P=0.011, respectively; **Figure 5B**). The overexpression levels of ALDOB in 7721 and HuH7 were shown in **Figure 5C**.

## Discussion

Liver cancer is a major public health concern worldwide. ^18^F-FDG PET/CT is the most widely used method to noninvasively evaluate tumor glucose metabolism by measuring ^18^F-FDG uptake. High FDG uptake is an indicator of poor prognosis. In this study, we investigated whether ^18^F-FDG PET/CT could predict the expression of ALDOB, and whether the combination of both factors could provide additional prognostic information on OS in HCC patients. Subsequently, ALDOB expression levels were negatively correlated with the tumor SUVmax of FDG uptake. Specifically, negative ALDOB expression and high SUVmax (≥3.9) are associated with poor disease prognosis in these patients, and SUVmax is recognized as an independent predictive factor for disease survival. Worse disease survival is suggested in HCC patients with negative ALDOB expression and high SUVmax.

ALDOB is a metabolic enzyme involved in glycolysis and fructose catabolism, and its expression is abnormal in various cancer types, including gastric cancer, colorectal cancer, clear cell renal cell carcinoma, and liver cancer (16, 17). Compared to normal tissues, ALDOB expression was more than seven-fold lower in gastric cancer tissues (18). Similarly, decreased ALDOB expression has been observed in clear cell renal cell carcinoma (19). In our study, ALDOB expression was absent in 25 of the 34 HCC patients. Decreased ALDOB protein levels were associated with tumor size, high TNM stage, and shorter OS in our cohort. These findings are consistent with those of previous studies. A large cohort of 313 patients was analyzed, and ALDOB downregulation was reported to be significantly correlated with aggressive characteristics, increased tumor size (>5 cm), and shorter recurrence-free survival and OS (6). Taken together, these results indicate that ALDOB may serve as a novel prognostic biomarker for HCC.

IHC analysis has been the gold standard for identifying gene expression status. However, obtaining tumor tissue by liver biopsy or resection is invasive. Several studies have suggested that ^18^F-FDG PET/CT, a noninvasive molecular imaging technique to detect malignant tumors, can predict gene expression status, such as LDHA in lung cancer and HER2 in gastric cancer (20, 21). Whether ^18^F-FDG PET/CT can predict ALDOB expression in HCC patients is unclear. In our study, we discovered that SUVmax, the most commonly used indicator in ^18^F-FDG PET/CT, was negatively correlated with ALDOB expression, with a correlation coefficient of -0.454. ROC curve analysis revealed an optimal SUVmax cutoff value to predict ALDOB expression in tumors was 4.15, with sensitivity and specificity of 76.2% and 88.9%, respectively. To our best knowledge, this is the first study to analyze the association between ^18^F-FDG uptake and ALDOB expression in patients with HCC. Assays on cultured cell lines were performed in this retrospective clinical analysis, which indicated that ALDOB overexpression led to decreased ^18^F-FDG uptake. However, the molecular mechanisms underlying the association between ^18^F-FDG accumulation and ALDOB status remain unclear. Many studies have found that the PI3K-AKT pathway plays a key role in regulating ^18^F-FDG accumulation in tumor cells (22, 23). He et al. found that ALDOB decreases glucose uptake by interacting with phosphorylated AKT and suppressing AKT activity (24). Additionally, ALDOB can also directly bind to insulin receptors, inhibit phosphorylation, and further dampen IR-PI3K-AKT signaling (25). These studies demonstrate that the negative association between ^18^F-FDG accumulation and ALDOB expression may be, in part, the result of the nonenzymatic function of ALDOB in HCC.

Furthermore, many previous studies have evaluated the prognostic value of ^18^F-FDG PET/CT for HCC and found that the SUVmax could provide a relevant biomarker for survival prediction (15, 26). In our study, an SUVmax cutoff of 3.9 was determined according to ROC analysis, and the OS for SUVmax ≥3.9 was worse. In line with our study, Han et al. retrospectively analyzed 298 HCC patients and found that SUVmax >3.5 can better predict disease-free survival and OS (27). Univariate Cox regression analysis showed that both AFP level and SUVmax were associated with OS in patients with HCC. Multivariate Cox analysis showed that SUVmax was the only recognized independent predictive factor for disease survival. Both ALDOB expression and SUVmax can predict OS in HCC patients. Whether the combined use of SUVmax and ALDOB provides valuable prognostic information for HCC patients is unclear. According to the Kaplan–Meier analysis, lack of ALDOB and high SUVmax were associated with worse prognosis and shorter OS.

The current study had some limitations. First, this retrospective study may have been affected by selection bias. Second, the number of patients included in the study was relatively small. Finally, the limited sample size made it difficult to validate our main findings. Therefore, more case series or prospective studies are needed to ratify the value of ALDOB expression and SUVmax in HCC.

In general, ALDOB mRNA and protein levels were significantly downregulated and correlated with tumor size, TNM stage, SUVmax, and prognosis in HCC patients. The correlation coefficient between SUVmax and the tumor ALDOB expression score was -0.454. Univariate and multivariate analyses showed that SUVmax is an independent prognostic risk factor for HCC. Patients with negative ALDOB expression and high SUVmax had worse OS. These results suggest that ^18^F-FDG PET/CT can predict ALDOB expression levels. Additionally, ALDOB expression and SUVmax may provide useful information for HCC prognosis and facilitate the development of ALDOB-mediated HCC therapies.

## Data availability statement

The original contributions presented in the study are included in the article/supplementary material. Further inquiries can be directed to the corresponding author.

## Ethics statement

The studies involving human participants were reviewed and approved by Shanghai Jiao Tong University affiliated with Renji Hospital. Written informed consent for participation was not required for this study in accordance with the national legislation and the institutional requirements.

## Author contributions

Study conception and design: GH and JLiu. Material preparation and data collection: WJ, JLi, MS, RZ and QW. Data analysis: QW, LZ and XY. Drafting of the manuscript: WJ and XY. Supervise the study, revise and finalize the manuscript: GH and JLiu. All authors contributed to the article and approved the submitted version.
